# Differential Regulation of Cysteinyl Leukotriene Receptor Signaling by Protein Kinase C in Human Mast Cells

**DOI:** 10.1371/journal.pone.0071536

**Published:** 2013-08-15

**Authors:** Vinay Kondeti, Ernest Duah, Nosayba Al-Azzam, Charles K. Thodeti, Joshua A. Boyce, Sailaja Paruchuri

**Affiliations:** 1 Department of Chemistry, University of Akron, Akron, Ohio, United States of America; 2 Department of Integrative Medical Sciences, Northeast Ohio Medical University, Rootstown, Ohio, United States of America; 3 Department of Medicine, Harvard Medical School, Boston, Massachusetts, United States of America; 4 Department of Pediatrics, Harvard Medical School, Boston, Massachusetts, United States of America; 5 Division of Rheumatology, Immunology and Allergy, Jeff and Penny Vinik Center for Allergic Disease Research, Brigham and Women’s Hospital, Boston, Massachusetts, United States of America; Fundação Oswaldo Cruz, Brazil

## Abstract

Cysteinyl leukotrienes (cys-LTs) are a group of lipid mediators that are potent bronchoconstrictors, powerful inducers of vascular leakage and potentiators of airway hyperresponsiveness. Cys-LTs play an essential role in asthma and are synthesized as well as activated in mast cells (MCs). Cys-LTs relay their effects mainly through two known GPCRs, CysLT_1_R and CysLT_2_R. Although protein kinase C (PKC) isoforms are implicated in the regulation of CysLT_1_R function, neither the role of PKCs in cys-LT-dependent MC inflammatory signaling nor the involvement of specific isoforms in MC function are known. Here, we show that PKC inhibition augmented LTD_4_ and LTE_4_-induced calcium influx through CysLT_1_R in MCs. In contrast, inhibition of PKCs suppressed c-fos expression as well MIP1β generation by cys-LTs. Interestingly, cys-LTs activated both PKCα and PKCε isoforms in MC. However, knockdown of PKCα augmented cys-LT mediated calcium flux, while knockdown of PKCε attenuated cys-LT induced c-fos expression and MIP1β generation. Taken together, these results demonstrate for the first time that cys-LT signaling downstream of CysLT_1_R in MCs is differentially regulated by two distinct PKCs which modulate inflammatory signals that have significant pathobiologic implications in allergic reactions and asthma pathology.

## Introduction

Cysteinyl leukotrienes (cys-LTs), comprising LTC_4_, LTD_4_ and LTE_4_. are potent bronchoconstrictors and mediators of pulmonary inflammation [1], [2]. They are derivatives of arachidonic acid generated by mast cells (MCs), eosinophils, basophils, macrophages, and myeloid dendritic cells [3]. LTC_4_ and LTD_4_ are very short-lived in vivo while LTE_4_ is stable, being the only cys-LT detected in biologic fluids and excreted in the urine [4]. Cys-LTs potentiate airway hyperresponsiveness (AHR) to histamine when administered by inhalation to human subjects [5]. Bronchoalveolar lavage (BAL) fluids collected from allergic human subjects after endobronchial challenge with allergen contain high levels of cys-LTs [6], pointing the role of cys-LTs in allergic inflammation. This role is confirmed by the fact that inhibitors of the type 1 G protein-coupled receptor (GPCR) for cys-LTs (CysLT_1_R) [7], [8] and inhibitors of cys-LT synthesis [9] are clinically efficacious for the treatment of asthma. Cys-LTs are also implicated in adaptive immunity and fibrosis [10], [11], [12]. Most of these cys-LT-mediated effects are thought to be induced through CysLT_1_R and a second GPCR, CysLT_2_R [13], [14], although the existence of additional receptors is likely based on findings in mice lacking both receptors [15], [16], [17]. Identification of signaling partners and mechanisms involved in the regulation of these receptors is crucial to gain insight into allergic inflammation.

MCs are stem cell factor (SCF)-dependent hematopoietic cells that are ubiquitously distributed throughout the body [18], [19] and initiate inflammatory responses to allergens and infectious agents. They play an important role in triggering exacerbations of asthma through the elaboration of several soluble inflammatory mediators including cys-LTs, histamine, serine proteases, multiple cytokines and chemokines. MCs not only generate cys-LTs, but also express both CysLT_1_R and CysLT_2_R [20], [21] and respond to LTC_4_, LTD_4_, and LTE_4_ with a range of functions. We have demonstrated earlier that stimulation of human cord blood-derived MCs (hMCs) and/or LAD2 cells with LTD_4_ potently induces calcium flux [21], [22] and cytokine generation [22], [23], each of which requires CysLT_1_R based on pharmacologic antagonism by MK571. hMCs also proliferate in response to LTD_4_, reflecting transactivation of c-kit by CysLT_1_R [24]. The relevance of cys-LTs to MC function is suggested by the observation that mice lacking the requisite terminal enzyme needed for cys-LT generation, leukotriene C_4_ synthase, show markedly reduced numbers of MCs in the airway mucosa following sensitization and challenge to allergen [12]. However, aside from the ability of LTD_4_ to transactivate c-*kit* [24] and for LTE_4_ to activate PPARγ [22] and induce the formation of large amounts of cytokines by a pathway involving the P2Y_12_ receptor [17], little is understood concerning the signaling mechanisms by which cysteinyl leukotriene receptors modulate the function of MCs.

Protein kinase C (PKC) refers to a family of phospholipid-dependent serine/threonine protein kinases that are activated by a number of extracellular stimuli including growth factors, adhesion, cytokines and GPCRs [25]. PKCs are involved in signal transduction associated with cell proliferation, differentiation, and apoptosis. At least eleven closely related PKC isozymes have been reported that differ in their structure, biochemical properties, tissue distribution, subcellular localization, and substrate specificity. They are classified as classical (α, β1, β2,γ), novel (δ, ε, η, θ, μ), and atypical (ξ, λ) isozymes depending on their requirement for the cofactors calcium, diacylglycerol (DAG) and phosphatidylserine (PS) [26], [27], [28]. PKCs are implicated in the negative regulation of LTD_4_-induced calcium signaling [29], [30]. Global pharmacological inhibition of PKCs was shown to inhibit LTD_4_-mediated CysLT_1_R internalization and desensitization resulting in enhanced phosphoinositide production and calcium flux [31]. This CysLT_1_R desensitization is shown to occur mainly through the phosphorylation of three serine residues (313–316) in the tail region of CysLT_1_R by PKCα [31]. In contrast, Thodeti et al., demonstrated that PKCε regulates LTD_4_-induced Ca^2+^ signal in intestinal epithelial cells [32]. Overall, it is not clear what specific isoforms are activated by cys-LTs in MCs or how they are involved in regulation of the LTD_4_-induced Ca^2+^ signal as well MC activation. In the present study, we investigated the specific PKC isoforms activated in MCs by cys-LTs and the role of each isoform in regulating cys-LT-induced MC responses. We show that both LTD_4_ and LTE_4_ activate PKCα and PKCε isoforms and that these isoforms regulate different signals down-stream of CysLT_1_R. Specifically, PKCα negatively regulates cys-LT-induced calcium flux, while PKCε positively regulates CysLT_1_R-mediated c-fos expression and MIP1β generation.

## Materials and Methods

### Reagents

LTD_4_, LTE_4_ and MK571 were purchased from Cayman Chemical. Fura-2 AM was from Molecular Probes, All phospho-specific antibodies were from Cell Signaling Technology, Total PKC antibodies were from Santa Cruz Biotechnology. Isoform specific siRNAs for PKCs were obtained from Dharmacon and MIP1β Elisa kit was from Endogen.

### Cell Culture

The LAD2 MC leukemia line [33] was a kind gift from Dr. Arnold Kirshenbaum, NIH. These cells were cultured in stempro-34 (Invitrogen) supplemented with 2 mM L-Glutamine (Invitrogen), Pen-strep (100 IU/ml) (Invitrogen) and SCF (endogen) (100 ng/ml). Cell culture medium was hemidepleted every week with fresh medium and 100 ng/ml SCF. Primary hMCs were derived from cord blood mononuclear cells cultured for 6–9 weeks in RPMI supplemented with SCF, interleukin IL-6, and IL-10 [34].

### Calcium Flux

LAD2 cells or hMCs (0.5–1×10^6^/sample) were washed and labeled with fura 2-AM for 30 minutes at 37°C. Cells were stimulated with the indicated concentrations of LTD_4_ and LTE_4_ and the changes in intracellular calcium were measured using excitation at 340 and 380 nm in a fluorescence spectrophotometer (Hitachi F-4500) as described earlier [22]. The relative ratios of fluorescence emitted at 510 nm were recorded and displayed as a reflection of intracellular calcium concentration. In some experiments, cells were pre-incubated with the PKC inhibitor GF109203X (GFX; 2 µM) for 30 minutes or with CysLT_1_R antagonist MK571 (1 µM) for 15 minutes before the stimulation with cys-LTs (500 nM).

### Cell Activation

LAD2 cells were either stimulated with 500 nM of LTD_4_ or LTE_4_ (unless specified otherwise), pre-treated with GFX (2 µM) for 30 minutes or MK571 (1 µM) and stimulated for 15 minutes for the phosphorylation of Erk and CREB or 1 h for the expression of c-fos or 6 h for the measurement of cytokines. The concentration of MIP1β (Endogen) was measured with ELISAs according to the manufacturer’s protocol [22]. Transfection of isoform specific siRNA smart pool constructs from Dharmocon (10 nM) were carried out using Silentfect transfection reagent (Biorad) for 48 h according to the manufacturer’s protocol.

### Cell Lysates and Western Blotting

After stimulation with the respective agonists, LAD2 cells (0.5×10^6^) were lysed with lysis buffer (BD Bioscience) supplemented with protease inhibitor cocktail (Roche) and phosphatase inhibitor cocktail (pierce). Immunoblotting was performed as described previously [35]. Briefly, lysates were subjected to 4–12% SDS-PAGE and transferred to PVDF membrane. Membranes were incubated with respective primary Phospho- and total antibodies diluted in 1x TBS, 5% dry milk, 0.1% Tween-20 (1∶1000) overnight at 4°C on shaker, and then with secondary antibody (peroxidase-conjugated anti-rabbit or anti-mouse). Western blot was incubated with ECL and the bands were visualized using imager (Protein Simple) and quantified using Image J (NIH).

### Statistics

Data are expressed as mean ± SD from at least three experiments except where otherwise indicated. Data were converted to a percentage of control for each experiment where indicated. Significance was determined using Student’s *t* test as well as one-way ANOVA followed by Tukey post-hoc analysis.

## Results

### Cys-LT-mediated Calcium Flux in Mast Cells is Negatively Regulated by PKC

**Figure 1:**
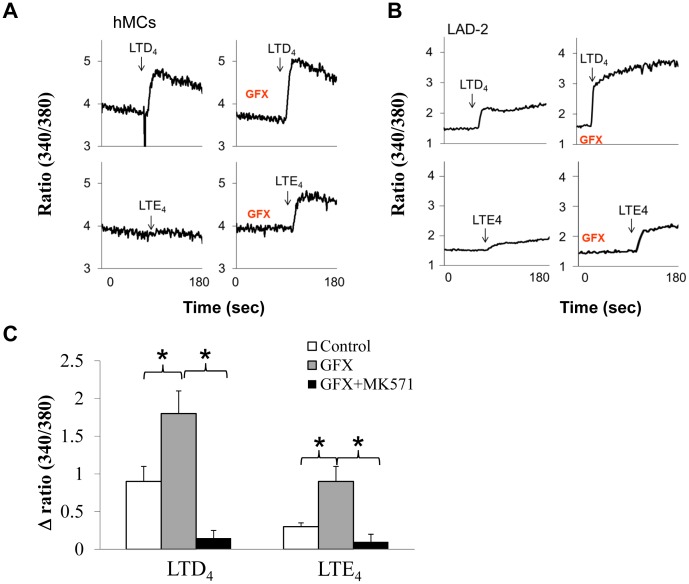
Effect of PKC inhibition on calcium signaling by LTD_4_ and LTE_4_ in LAD2 cells. Calcium transients from hMC (A) and LAD2 cells (B) with cys-LTs (500 nM) in presence or absence of PKC inhibitor, GFX (2 µM) (C) Quantitative analysis of calcium influx from A and B and the effect of MK571 (1 µM) on the enhanced calcium flux with GFX pre-treatment. The data shown are±SD of three experiments. The significance was tested using Student’s t-test as well as one-way ANOVA followed by Tukey post-hoc analysis. P<0.05. NS = non-significant.

We have reported earlier that cys-LTs, especially LTD_4,_ potently induces calcium flux in primary hMCs [21] and also in LAD2 cells [22]. This signal was sensitive to inhibition by MK571, implying a requirement for CysLT_1_R or a CysLT_1_R-like GPCR in this signaling event. CysLT_1_R undergoes ligand-induced desensitization and internalization in heterologous cell systems and these processes are uniquely dependent on PKC [31]. Based on these observations, we sought to determine if PKCs have a role in controlling cys-LT-dependent calcium flux in MCs. Both hMCs and LAD2 cells were pre-treated with GF109203X (GFX), a global PKC inhibitor, and its effect on LTD_4_ or LTE_4_ stimulation was evaluated. In the absence of GFX, LTD_4_ (500 nM) potently stimulated calcium flux in both cell types, but LTE_4_ (500 nM) only caused minimal calcium flux. However, GFX treatment markedly potentiated LTD_4_ and LTE_4_-mediated calcium fluxes in both cell types (Fig. 1 A, B). Importantly, a specific antagonist of CysLT_1_R, MK-571, completely abolished both LTD_4_ and LTE_4_-mediated calcium fluxes in the presence of GFX (Fig. 1C). These observations suggest that the strength of calcium signaling through CysLT_1_R is negatively regulated by PKCs, probably through the desensitization of the receptors [31], [36].

### PKCs are Required for cys-LT-mediated Phosphorylation and Expression of c-fos

**Figure 2:**
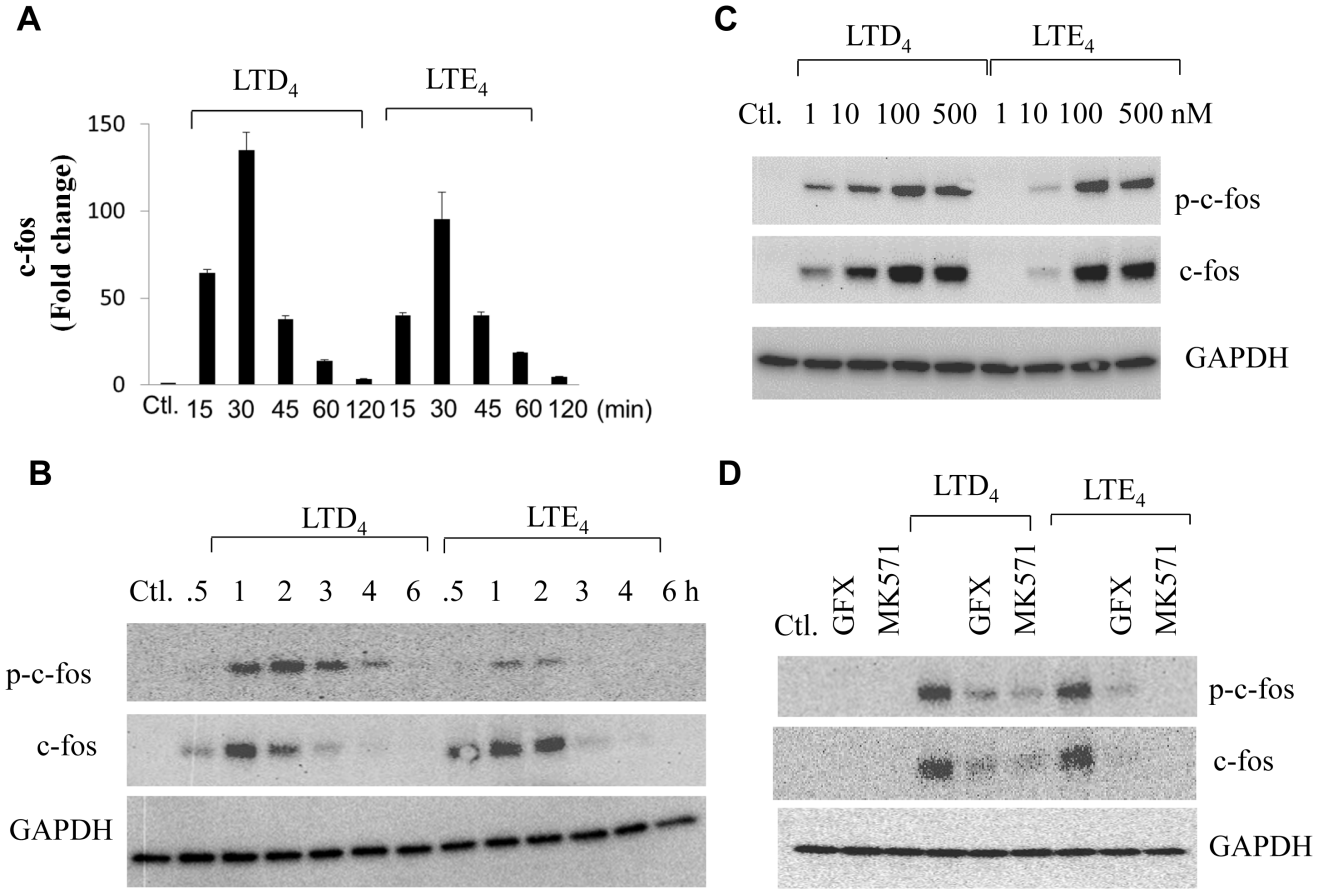
LTD_4_ and LTE_4_-induced phosphorylation and expression of c-fos in LAD2 cells and the effect of PKC inhibition. Relative levels of c-fos transcript (A) upon treatment with 500 nM of LTD_4_ and LTE_4_, c-fos phosphorylation and expression with LTD_4_ or LTE_4_ (500 nM) for indicated period (B), Dose response (C), Pre-treated with GFX (2 µM) or MK571 (1 µM) (D), stimulated with 500 nM of cys-LTs and analyzed by western blotting. Blots were stripped and blotted for GAPDH. The data shown are representative of three separate experiments.

In rat basophilic leukemia (RBL) cells, Ng et al., demonstrated that disrupting CysLT_1_R desensitization by PKC inhibitors can lead to enhanced LTC_4_-induced calcium influx, but prevents up-regulation of c-fos expression through the CRAC channels. Along these lines, we first checked if stimulation of MCs with LTD_4_ and LTE_4_ induced c-fos expression (Fig. 2). We found that both LTD_4_ and LTE_4_ induced robust activation of c-fos at the transcript level as well as at the protein level. Surprisingly, the induction of c-fos transcript was maximum at 30 minutes, while the protein induction was as early as 30 minutes with peak expression at 1 h and then slowly began to decline after stimulation with either LTD_4_ or LTE_4_ (Fig. 2A, B). To determine the potency of cys-LTs to induce the expression of c-fos, we treated LAD2 cells with various concentrations of LTD_4_ and LTE_4_ and analyzed phosphorylation and induction of c-fos (Fig. 2C). LTD_4_ caused c-fos induction at doses as low as 1 nM while LTE_4_ evoked similar response at relatively higher concentrations (100 nM and 500 nM). On average, we found that 500 nM concentration of cys-LTs evoked the best response of all the experiments performed and hence we stimulated cells with 500 nM of cys-LTs in all the concurrent experiments. Also, we observed that the pattern of phosphorylation as well as expression of c-fos were similar with both LTD_4_ and LTE_4_, suggesting that cys-LTs not only induced the expression of c-fos but also activated c-fos. We then asked if cys-LT-induced c-fos expression and activation are sensitive to PKC inhibition and are mediated through cysLT_1_R. Both LTD_4_ and LTE_4_-induced c-fos activation as well as expression was inhibited by GFX as well as MK571 (Fig. 2D). These results suggest that though PKCs negatively regulate cys-LT-mediated calcium flux, but are required for cys-LT-mediated c-fos phosphorylation/expression.

### MIP1β Generation by cys-LTs is Positively Regulated by PKCs

**Figure 3:**
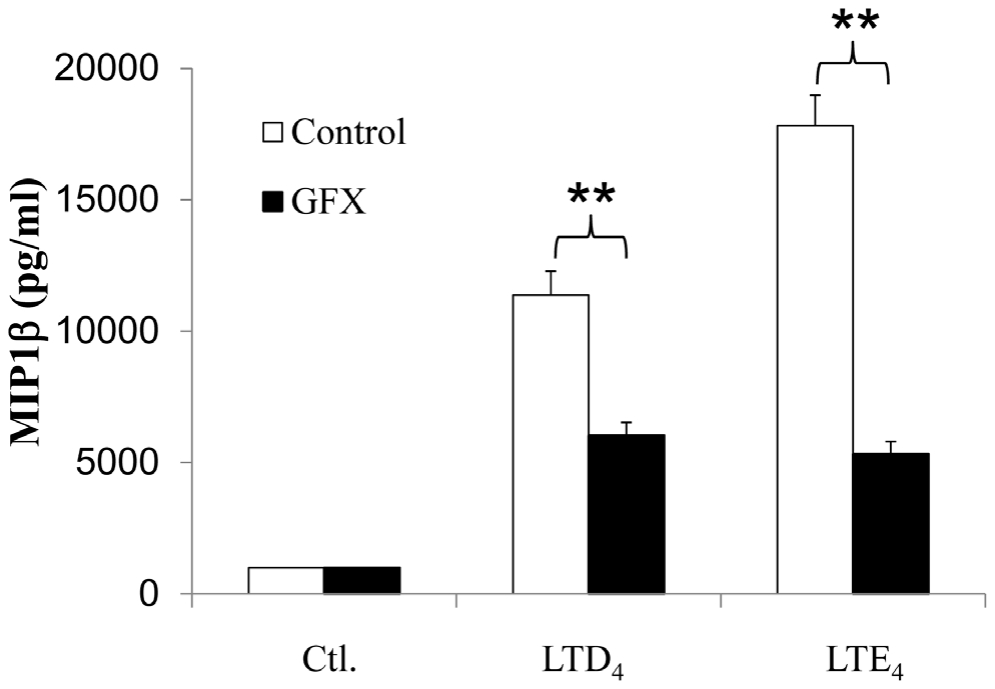
Involvement of PKC in LTD_4_ and LTE_4_-induced MIP1β secretion in LAD2 cells. LAD2 cells were stimulated with 500 nM of LTD_4_ or LTE_4_ for 6 h in presence or absence of GFX (2 µM). The generation of MIP1β was analyzed from the culture supernatant using MIP1β-specific ELISA. Data shown are±SD of three independent experiments. ** P<0.001. NS = non-significant.

Next, we investigated the effect of PKC inhibition on other cys-LT-induced MC functions. We have shown earlier that cys-LTs are capable of potently activating inflammatory chemokine, MIP1β in MCs [22]. Hence, we asked if PKCs play a role in cys-LT-induced inflammatory responses such as MIP1β production in MCs. To determine this, LAD2 cells were pre-treated with GFX with or without cys-LT stimulation and MIP1β was measured in the supernatants. As reported earlier [22] and shown in Fig. 3, both LTD_4_ and LTE_4_ potently induced MIP1β generation. Importantly, unlike calcium flux, MIP1β induction by both the agonists was significantly blocked by PKC inhibition with GFX (Fig. 3). These findings suggest the PKCs differentially regulate cys-LT-induced calcium influx and gene expression in MCs, possibly via activation of distinct isoforms of PKCs.

### PKCs do not Effect cys-LT-activated ERK, or CREB Pathways

**Figure 4:**
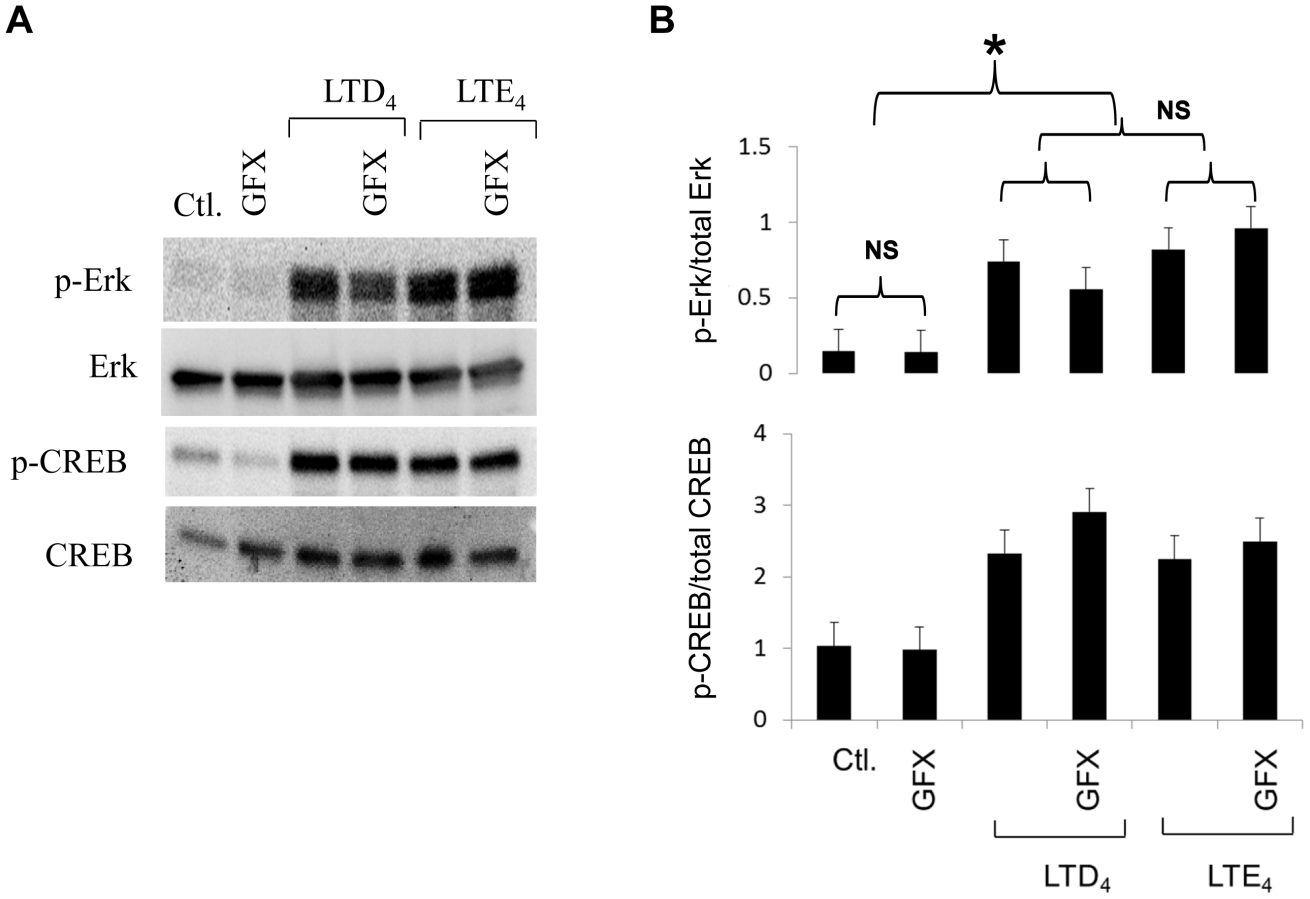
Phosphorylation of Erk and CREB by LAD2 cells in response to LTD_4_ and LTE_4_ and the role of PKC in mediating these effects. (A) phosphorylation as well as total expression of Erk and CREB proteins by Western blotting in cell lysates of LAD2 cells pre-treated with GFX (2 µM) and stimulated with 500 nM of LTD_4_ and LTE_4_ respectively for 30 minutes (B) Quantitative analysis. The shown data represents±SD of three separate experiments. *P<0.05. NS = non-significant.

We have shown earlier that cys-LTs activate ERK and CREB [22] and we sought to investigate if all cys-LT-induced effects are mediated through PKCs. To our surprise, PKC inhibition by GFX had no significant effect on the phosphorylation or the expression of ERK and CREB by cys-LTs (Fig. 4). These results suggest that cys-LTs have potential to modulate MC function, both dependent as well as independent of PKCs.

### PKC Profile in MCs and Identification of cys-LT-responsive PKC Isoforms

**Figure 5:**
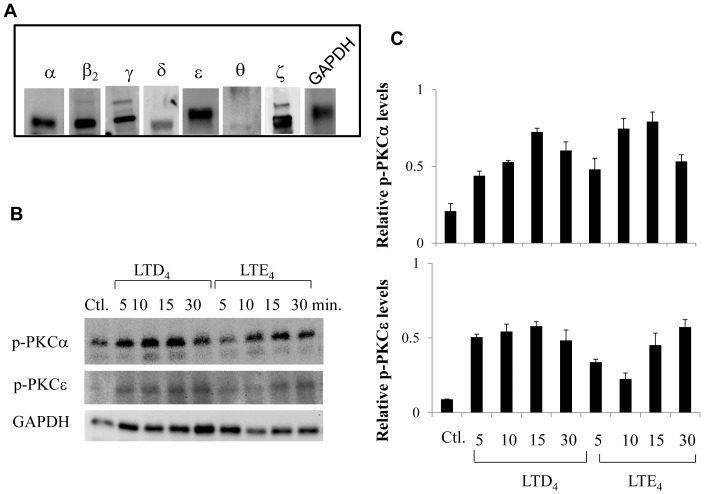
Identification of possible PKC isoform(s) activated by LTD_4_ and LTE_4_ in LAD2 cells. (A) Expression of PKC isoforms and GAPDH (B) phosphorylation of PKCα and PKCε stimulated with 500 nM of LTD_4_ or LTE_4_ for the indicated times (C) Quantitative analysis of relative phospho-PKC levels in LAD2 cells. Data shown are±SD of three separate experiments.

To determine which of the PKC isoforms mediate cys-LT signaling responses, we first characterized the expression of different isoforms of PKCs in MCs including classical PKCs (α, βI, βII, γ), novel PKCs (δ, ε, η, θ), and atypical PKCs (ζ, ι/λ, μ) by Western blotting. We found that MCs express PKC α, βII, γ, δ, ε, θ and ζ isoforms (Fig. 5A) and not βI, η, ι/λ, μ (data not shown). We next asked which of the expressed PKC isoforms are activated by cys-LTs. Cys-LT responsive PKC isoforms were determined by analyzing the phosphorylation of individual PKC isoforms in response to cys-LTs using phospho-specific antibodies. We found that PKCα and PKCε are phosphorylated by both LTD_4_ and LTE_4_ in a time dependent manner (Fig. 5B, C), but not PKC βII, γ, δ, θ and ζ isoforms (data not shown). Phosphorylation of both PKCα and PKCε in response to cys-LTs was rapid and transient reaching a peak at 15 minutes and started to decline after 30 minutes. The peak LTE_4_-induced phosphorylation of PKCε, but not of PKCα was more gradual than that induced by LTD_4_. The small inhibition in the phosphorylation of PKCε that we observed at 10 minutes compared to 5 minutes in response to LTE_4_ is not statistically significant.

### PKCα Negatively Regulates cys-LT Mediated Calcium Flux While PKCε is Essential for MIP1β Generation by cys-LTs

**Figure 6:**
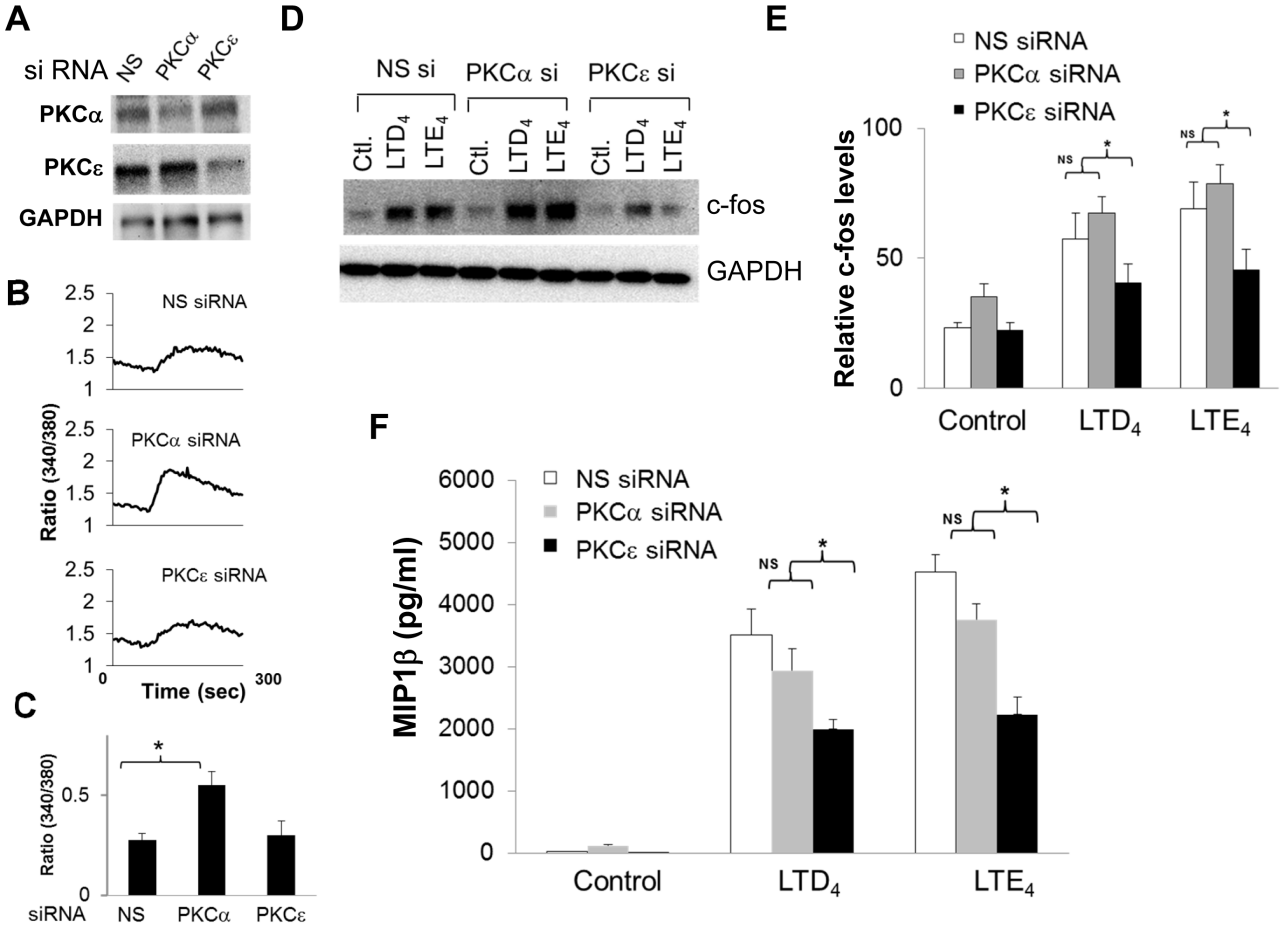
Effect of siRNA-mediated knockdown of PKCα and PKCε on cys-LT-mediated calcium, c-fos and MIP1β generation by LAD2 cells. PKCα and PKCε isoforms were Knocked down using specific siRNAs against PKCα and PKCε (10 nM). Non-specific (NS) siRNA was used as a control. The siRNA treated cells were analyzed for (A) the expression of PKCα, PKCε and GAPDH, (B, C) LTD_4_ (500 nM)-induced calcium influx, and quantitative analysis, (D, E) cys-LT-induced c-fos expression, (F) MIP1β production. Cells were treated with 500 nM of LTD_4_ and LTE_4_ for 5 minutes (calcium flux), 1 h (c-fos expression) and 6 h (MIP1β). Data shown are±SD of three separate experiments. *P<0.05, **P<0.001.

After determining that LTD_4_ and LTE_4_ both activated PKCα and PKCε in MCs, we investigated the specific roles of PKCα and PKCε in cys-LT-mediated calcium flux, c-fos expression and MIP1β production (Fig. 6). To determine this, we first knocked down PKCα and PKCε isoforms in LAD2 cells by transfecting isoform specific siRNAs (10 nM) against PKCα and PKCε. As a control, we transfected cells with a non-specific siRNA pool. Transfection of MCs with PKCα and PKCε siRNAs significantly down regulated PKCα and PKCε expression (40.0±4.3% and 41.5±9.2% down regulation), respectively (Fig. 6A). Down regulation of PKCα with PKCα siRNA did not have any significant effect on the expression of PKCε and vice versa (data not shown). We then assessed cys-LT mediated calcium influx, c-fos phosphorylation, expression and MIP1β generation in these cells. Calcium measurements revealed that knock down of PKCα induced a significant two fold increase in LTD_4_-induced peak calcium influx in MCs (Fig. 6B, C). We did not detect any change in calcium flux induced by LTD_4_ in PKCε knocked-down MCs suggesting that PKCα is the key isoform involved in the negative regulation of cys-LT induced calcium flux. On the other hand, knockdown of PKCε attenuated both LTD_4_ and LTE_4_-induced c-fos expression (Fig. 6D, E) and phosphorylation (data not shown). Knock down of PKCε also attenuated cys-LT-induced MIP1β production in MCs (53% and 55% respectively) (Fig. 6F). Transfection with control siRNAs did not affect LTD_4_ and LTE_4_-induced c-fos expression or MIP1β generation. Although PKCα knock down marginally inhibited MIP1β generation, this signal is not significantly different from control siRNA.

## Discussion

**Figure 7:**
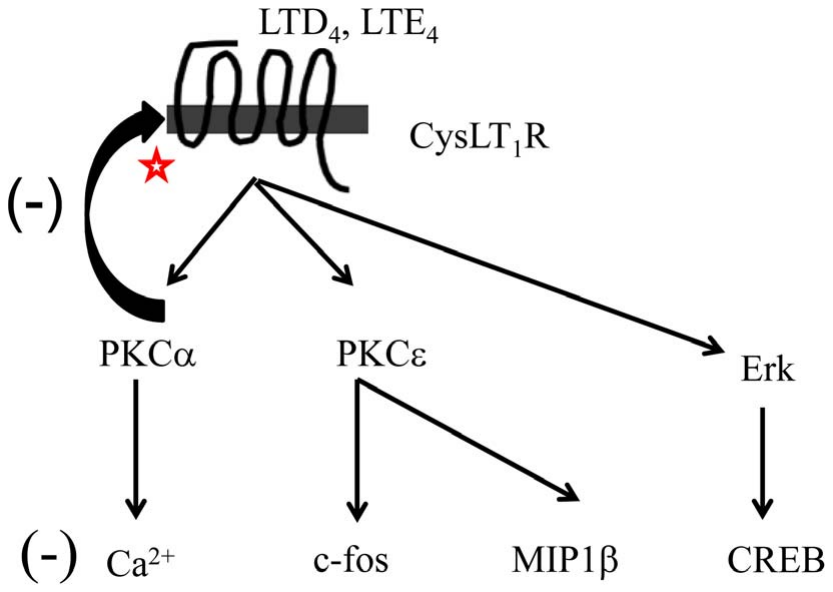
A model depicting PKC regulated cys-LT signaling in MCs. Hypothetical mechanism(s) depicting cys-LT-mediated signaling in MCs by PKCs. CysLT_1_R activation by LTD_4_ or LTE_4_ activates PKCα and PKCε. PKCα desensitize CysLT_1_R by phosphorylating the receptor and negatively regulating the calcium flux. On the other hand, PKCε activation by CysLT_1_R activates c-fos expression, MIP1β production. Cys-LTs also activate Erk and CREB independent of PKCs.

In the present study, we demonstrate that cys-LTs activate two isoforms of protein kinases, PKCα and PKCε and that these two isoforms differentially regulate cys-LT-mediated MC function. PKCε is essential for cys-LT-mediated c-fos expression and MIP1β generation, while PKCα negatively regulates cys-LT-induced calcium flux (schematic, Fig. 7). Surprisingly, PKCs appear to be dispensable for expression and activation of ERK and CREB.

MCs are relevant cellular effectors of asthma and other allergic diseases, and cys-LTs are pertinent mediators of the same processes [37]. The mechanisms that control cys-LT-dependent biological responses are of considerable pathobiologic and clinical interest in both allergic and non-allergic disease [38]. We have previously demonstrated that cys-LTs induce robust calcium flux in hMCs [21], [23] and LAD2 cells via CysLT_1_R (based on pharmacologic interference using selective antagonists) [22]. We have shown earlier that MK571 specifically blocks calcium flux and Erk phosphorylation in CHO cells expressing CysLT_1_R, but not CysLT_2_R suggesting its specificity [22]. MK571 is also reported to have inhibitory activity against MRP1 [39]. Further, it was shown that MK571 treatment increased intracellular LTC_4_ concentration in eosinophils and modulate IL-4 levels from preformed vesicles via a putative intracellular CysLT receptor [40]. However, cys-LT-induced inflammatory mediator production in MCs require de novo transcriptional and translational mechanisms and no such putative intracellular CysLTR has been identified. Therefore, we believe that the observed inhibitory effects of MK571 are mostly directed at CysLT_1_ receptor on the plasma membrane. In the current study, we elucidate that pharmacological inhibition of PKCs followed by stimulation of cells with cys-LTs resulted in significant augmentation of calcium flux in MCs. This finding is consistent with desensitization of CysLT_1_R by PKCs reported in other cell systems. Crooke and colleagues observed that LTD_4_ activates PKC, and the same research team [29], [41] noted that inhibitors of PKC increased the mobilization of Ca^2+^ induced by LTD_4_ in the leukemic cell line RBL-1 using pharmacological activators and inhibitors. Winkler et al. [30] have reported that the broad PKC inhibitor staurosporine potentiated the LTD_4_-induced Ca^2+^ signal in differentiated U-937 cells. In COS-1 cells overexpressing CysLT_1_R, pharmacological inhibition of PKC activity was shown to enhance calcium mobilization stimulated by LTD_4_ [31]. However the exact molecular mechanism(s) underlying this process are not well known.

Enhanced receptor activation is usually translated into increased receptor function. Relief of PKC-mediated desensitization of endogenous CysLT_1_R augments multiple LTD_4_-stimulated cellular functions, with associated increases in intracellular signaling events [42]. However, while our data indicate that PKC inhibition augmented cys-LT-induced calcium signaling, we also found that it suppressed cys-LT-induced c-fos expression and chemokine secretion. Activation of c-fos by LTD_4_ has been reported previously in HEK cells expressing CysLT_1_R [43]. Recently, Ng et al., reported that LTC_4_-mediated CysLT_1_R is desensitized by PKC-dependent phosphorylation and that prevention of this signaling by PKC inhibition led to loss of calcium-dependent gene expression, despite potentiation of Ca^2+^ release [36]. This signal was proposed to delay the activation of CRAC channels resulting in the decreased c-fos expression. In the present study using LAD2 cells, we observed that both LTD_4_ and LTE_4_ significantly increased the expression of c-fos, consistent with the earlier study [36]. Our data demonstrate that LTD_4_ and LTE_4_ also induce c-fos phosphorylation. This increase in phosphorylation and expression of c-fos is mediated through an MK-571 sensitive CysLTR and PKC. Since cys-LTs activate both Erk and CREB [22], we investigated if PKC inhibition altered cys-LT-mediated phosphorylation of these signaling molecules. Although cys-LTs robustly enhanced phosphorylation of Erk and CREB, inhibition of PKCs surprisingly had no effect on this signal. These findings suggest that modulation of PKC activity may couple CysLTR signaling to distinct signaling pathways. It is also possible that at least some of the PKC-independent signaling events may occur through receptors other than CysLT_1_R.

Despite the fact that cys-LT-mediated calcium signaling was enhanced by global PKC inhibition (Fig. 1), c-fos expression and MIP1β generation was substantially suppressed. While this finding could reflect a requirement for CysLT_1_R receptor desensitization to facilitate gene induction as suggested by the Ng et al., it also suggested that cys-LTs activate more than one PKC isoform in MCs. Indeed, we found that MCs express PKC α, βII, γ, δ, ε, θ and ζ isoforms but only PKCα and PKCε were phosphorylated in response to cys-LTs. Notably, we found that PKCα knockdown significantly augments calcium flux, but has little effect on cys-LT-induced c-fos and MIP1β production. However, knockdown of PKCε significantly attenuated cys-LT-induced c-fos phosphorylation, expression and MIP1β production without altering calcium flux. Activation of PKCε by cys-LTs has been showed in other systems [17], [32], [44] as well. Interestingly, PKCε was shown to be essential for LTD_4_-induced calcium signal in intestinal epithelial cells, suggesting that coupling of cys-LTs to signaling events is regulated in a cell type-specific manner. In conclusion, our study identifies specific isoforms of PKCs, PKCα and PKCε that are activated by cys-LTs and differentially regulate distinct MC functions, critical for the progression and pathology of asthma. Understanding the signaling and players involved in CysLTR regulation can be useful in identifying better therapeutic targets for inflammatory asthma and allergic diseases.
